# A Systematic Review of Whether Physical Activity Prevents Colorectal Cancer in Older Adults

**DOI:** 10.7759/cureus.95234

**Published:** 2025-10-23

**Authors:** Syeda Samrina Jannat, Zakaria Mohammed, Adam Grice, Daniel Jones

**Affiliations:** 1 Emergency Medicine, Calderdale and Huddersfield NHS Foundation Trust, Huddersfield, GBR; 2 Pediatrics, Midland Metropolitan University Hospital, Smethwick, GBR; 3 National Institute for Health and Care Research (NIHR), Leeds Institute of Health Sciences, University of Leeds, Leeds, GBR; 4 Family Medicine, North Cumbria Integrated Care, Carlisle, GBR

**Keywords:** cancer prevention, colorectal cancer, older adults, physical activity, risk reduction

## Abstract

Background: An ageing population and an increasing cancer incidence are both public health challenges. Previous literature has established an association between physical activity (PA) and a decreased risk of colorectal cancer (CRC). However, studies pertaining to older adults are limited.

Objective: This systematic review aims to investigate whether PA prevents CRC in adults aged ≥65 years. The secondary objective is to examine whether CRC risk varies with type, intensity, and/or frequency of PA.

Methods: A systematic review was conducted in accordance with Preferred Reporting Items for Systematic Reviews and Meta-Analyses (PRISMA) 2020 guidelines. Ovid MEDLINE, Embase, Cumulative Index to Nursing and Allied Health Literature (CINAHL), and SPORTDiscus were searched from inception to February 2025. Reference lists of previous studies were also reviewed. Eligible studies included adults aged ≥65 years, assessed the impact of PA on CRC diagnosis, reported empirical outcome data, and were published in English. Studies involving non-human participants or lacking age-specific outcomes were excluded. Methodological quality was assessed using the Mixed Methods Appraisal Tool (MMAT). A narrative synthesis was conducted to analyse the included studies.

Results: The search identified 3,604 papers, of which three studies met the inclusion criteria. Overall, there was no consistent association between PA and CRC risk in older adults. One study found no association, a second suggested PA was protective at the highest activity levels, while a third indicated high levels of PA may be harmful. There was also insufficient data to draw any conclusion regarding the secondary outcome.

Limitations: This review was limited by the small number of eligible studies (n = 3) and heterogeneity in activity types and frequency, as well as study durations, which prevented direct comparison. The limited number of studies may reflect the underrepresentation of older adults in research, often due to comorbidities and lower participation rates in this population.

Conclusion: Evidence on whether PA prevents CRC in older adults remains inconclusive. Further research focusing on PA type, intensity, and/or frequency is needed to guide age-appropriate public health recommendations.

## Introduction and background

Colorectal cancer (CRC) is the fourth most common cancer in the UK, and the third most diagnosed globally [1,2]. It is a leading cause of cancer death; annually, it accounts for over 16,000 deaths in the UK [3]. CRC risk increases with advancing age, with the peak incidence observed among adults aged between 85 and 89 [1]. The number of adults aged ≥65 years is often taken to be an indicator of population ageing; by 2028, around 20% of the UK population will fall into this age group [4]. Additionally, it is estimated that around three million adults will be over the age of 85 by 2043 [5]. 

A number of risk factors are associated with CRC, including cigarette smoking, diet, alcohol, and low physical activity (PA) levels [6]. Low PA engagement results in around 5% of CRC diagnoses [7,8]. Any movement of the body through the use of skeletal muscles that leads to energy being expended can be deemed as PA [9,10]. Importantly, PA, exercise, and sports have separate definitions. Repeated, planned bouts of PA can be referred to as exercise, such as swimming and running; examples of sports include basketball, volleyball, and football, which are forms of exercise and PA, but require rules to be adhered to [9-11]. In this study, PA will include exercise and sports, along with occupational, recreational, and household activities [9,10]. The World Health Organization (WHO) reports that 25% of men and 33% of women are not sufficiently active [9]. The association between PA and CRC has been well-documented in the literature. A systematic review and meta-analysis reported a 21% colon cancer risk reduction among very active participants (≥8000 metabolic equivalent (MET) minutes per week) compared to those who were not as active (<600 MET minutes per week) [12]. A 24% decreased colon cancer risk was reported in another meta-analysis [13]. Despite the benefits of PA appearing to be clear, there is much less research into the length and nature required.

Research about PA and CRC prevention in older adults is limited. The same decreased risk was noted by Chao et al. [14] in individuals aged 50-74, whom they referred to as an older population, as well as by Howard et al. [15] in individuals aged 50-71. Cohen et al. [16] agreed with this reduced risk in ≥65-year-olds, though CRC was among a number of cancers included in this study. This was supported by a previous paper, which included some analysis for ≥65-year-olds [17]. The different age groups in the aforementioned studies emphasises how there is no universally accepted definition of what constitutes an older population. An older person is frequently defined as being 65 or above, as agreed by the WHO [18]. PA levels often decline with age, and this contributes to greater disease and mortality risk [19]. The reasons for reduced PA engagement in this age group include breathlessness, fear of falling, absence of energy and motivation, and pain in joints [20]. These represent the potential difficulties of promoting PA in this population.

The protective role of PA against CRC is well-established, but its effectiveness in older adults remains unclear. Although CRC is more prevalent in older adults, most existing studies have focused on younger or mixed-age populations, leaving this group underrepresented in the literature. As the proportion of older adults continues to rise, addressing their health needs is becoming increasingly urgent. To date, no systematic review has specifically evaluated PA in the prevention of CRC among adults aged ≥65 years. 

Aim and objectives 

This study aims to investigate whether PA prevents CRC in older adults. The WHO definition of an older person will be used (aged ≥ 65 years) [18]. The exposure and outcome variables are PA and CRC diagnosis, respectively. The secondary outcome is to investigate whether CRC risk varies by type, intensity, and/or frequency of PA.

## Review

Methods

Protocol and Registration

This review was conducted in accordance with the Preferred Reporting Items for Systematic Reviews and Meta-Analyses (PRISMA 2020) guidelines [21]. The protocol was registered on the PROSPERO international prospective register of systematic reviews (CRD42021222222) [22]. 

Search Strategy

A literature search was conducted on Ovid MEDLINE, Embase, CINAHL, and SPORTDiscus, covering the dates from which each database was established to 26 December 2020 (MEDLINE, 1946; EMBASE, 1996; CINAHL, 1981; SPORTDiscus, 1949). An updated search was later conducted covering the date from which each database was established to 15 February 2025.

Keywords related to the following components were searched using the advanced search function: older adults, colorectal cancer, and physical activity. A complete list of search terms is shown in Table 1. Active Lives survey data tables from Sport England were used to help obtain search terms pertaining to PA [23].

Search terms were also mapped to subject headings, and Medical Subject Headings (MeSH) terms were applied, where appropriate. To substitute different endings of key terms, the truncation asterisk symbol was used (*). The OR function was used to combine synonyms for each of the three individual components, and the AND function was used to combine overall search terms. To identify any further papers, the reference lists of existing relevant literature were also reviewed.

**Table 1:** Search terms To substitute different endings of key terms, the truncation asterisk symbol was used (*)

Colorectal cancer	Physical activity	Older adults
1. Colorectal cancer*	1. Physical activit*	1. Older adult*
2. Colorectal malignanc*	2. Exercis*	2. Older people
3. Colorectal carcinoma*	3. Sport*	3. Older person*
4. Colorectal aden*	4. Athletic*	4. Older m#n
5. Colorectal tumor*	5. Team sport*	5. Older wom#n
6. Colorectal metasta*	6. Physical train*	6. Elderly
7. Colorectal oncolog*	7. Physical fitness	7. Frail*
8. Colorectal neoplas*	8. Fitness	8. Geriatric*
9. Bowel cancer*	9. Recreational activit*	9. Senior*
10. Colon cancer*	10. Occupational activit*	10. 65 year*
11. Rectal cancer*	11. Household activit*	11. Sixty five year*
	12. Leisure time physical activit*	12. Sexagenarian
	13. Leisure activit*	13. Septuagenarian
	14. Yoga	14. Octogenarian
	15. Pilates	15. Nonagenarian
	16. Running	16. Centenarian
	17. Runner*	17. Gerontolog*
	18. Sprint*	18. (older adj (adult? or m#n or wom#n or person? or people))
	19. Walk*	
	20. Step*	
	21. Treadmill	
	22. Power walk*	
	23. Trek*	
	24. Jog*	
	25. Climb*	
	26. Gym*	
	27. Swim*	
	28. Danc*	
	29. Basketball	
	30. Cycl*	
	31. Bik*	
	32. Box*	
	33. Football	
	34. Soccer	
	35. Rugby	
	36. Golf*	
	37. Martial Art*	
	38. Tennis	
	39. Badminton	
	40. Volleyball	
	41. Weight train*	
	42. Wrestl*	
	43. Aerobic*	
	44. Zumba	
	45. Garden*	
	46. Cardio*	
	47. Strength train*	
	48. Resistance train*	
	49. “High intensity interval train*”	
	50. Triathlon	
	51. Weight lift*	
	52. Karate	
	53. Tai chi	
	54. Judo	
	55. Mountaineering	
	56. Bowls	
	57. Combat sport*	
	58. Water sport*	
	59. Cricket	
	60. Exercise machine*	
	61. Hockey	
	62. Canoe*	
	63. Fitness class*	
	64. Racket sport*	
	65. Abseiling*	
	66. Archery	
	67. Baseball	
	68. Softball	
	69. Hill walk*	
	70. Mountain walk*	
	71. Sail*	
	72. Shooting	
	73. Squash	
	74. Ski*	

Eligibility Criteria

The inclusion criteria was as follows: (i) included adults aged ≥65 years; (ii) assessed the impact of PA on CRC diagnosis; (iii) focused on CRC prevention; (iv) reported empirical outcome data (randomised trials, cross-sectional, cohort, or case-control studies); (v) involved human participants only; and (vi) English language only. 

Selection Process and Data Extraction 

Search results were exported into EndNote X9 (Clarivate Analytics). Titles and abstracts were screened independently and blindly by two reviewers using the eligibility criteria. Full texts of potentially eligible articles were then independently assessed by the same reviewers. Discrepancies at any stage were resolved through discussion with two additional reviewers. 

The included study data were recorded on a predefined extraction template. Data were extracted blindly and independently by two reviewers. The extraction template included the following: (i) details of the paper (title, year, author, journal, volume, issue, pages); (ii) details relevant to this study (study design, number of subjects, and the age range, PA type/intensity/frequency); and (iii) results (outcome measures).

Risk of Bias Assessment

Methodological quality was assessed independently by two reviewers using the Mixed Methods Appraisal Tool (MMAT) [24]. It was agreed that no studies would be excluded based on this quality assessment. For each study type, the number of criteria met was divided by five and multiplied by 100 to generate a percentage score [25].

Synthesis Methods

Due to heterogeneity in study designs, exposure definitions, and outcome measures, included studies were analysed using a narrative synthesis approach. 

Certainty of Evidence

In this review, conducting a formal Grading of Recommendations, Assessment, Development and Evaluation (GRADE) assessment was not feasible due to the small number of eligible studies and the substantial variability across study designs, the types and levels of PA examined, and the way outcomes were originally reported. Although all studies met the predefined inclusion criteria, these differences limited comparability and precluded systematic application of the GRADE domains. Certainty of evidence was therefore considered narratively, taking into account study quality, consistency, and precision of results. 

Results

Study Selection 

**Figure 1:**
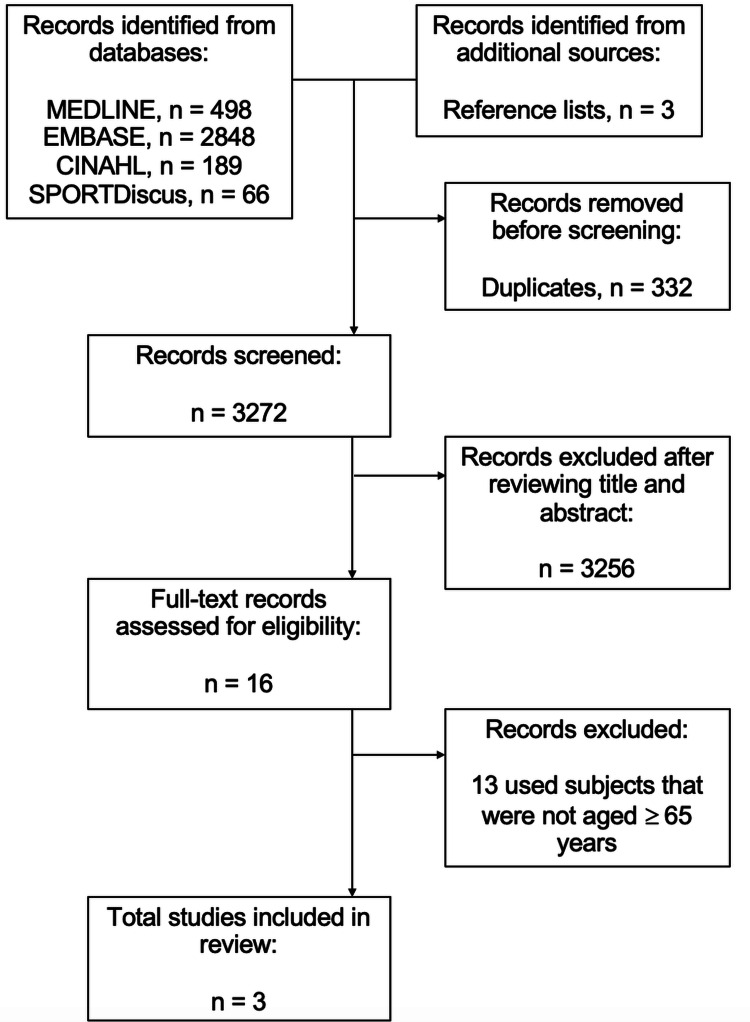
PRISMA flow chart PRISMA: Preferred Reporting Items for Systematic Reviews and Meta-Analyses

The updated literature search (2025) identified 3,604 papers. After removal of duplicates, 3,272 records remained. Screening of titles and abstracts excluded 3,256 records, leaving 16 for full-text review. Of these, three papers met the inclusion criteria. These were the same three studies that had met the inclusion criteria during the original 2020 search. A detailed flow diagram of the search and selection process is shown in Figure 1 (PRISMA flow chart).

Study Characteristics 

The three studies were observational in design. Cohen et al. [16] included a proportion of data pertaining to colon cancer; Slattery et al. [17] and Vena et al. [26] included a proportion of data relating to adults aged 65 and above.

Table 2 summarises some characteristics of the studies included. Odds ratios (OR) were reported by Slattery et al. [17] and Vena et al. [26]. However, Cohen et al. [16] reported hazard ratios (HR); the relevant data were converted to OR by contacting the authors. 

**Table 2:** Summary of study characteristics

Author	Year	Study design	Age (years)	Sex	Sample size	Method of assessment
							Cohen et al. [16]	2020	Cohort	69-78	Male and female	1542	Interview and questionnaire
Slattery et al. [17]	2003	Case-control	30-79	Male and female	5047	Interview and questionnaire
Vena et al. [26]	1985	Case-control	30-79	Male	1917	Interview and questionnaire

Risk of Bias Assessment 

All three studies met the five quality criteria, achieving overall scores of 100% (Table 3). Each study was therefore judged to be at low risk of bias. 

**Table 3:** Risk of bias assessment of included studies using MMAT MMAT: Mixed Methods Appraisal Tool Criteria summarised from the MMAT [24] for quantitative non-randomised studies. ✔ indicates the criterion was met

Author	Representative sample	Appropriate measurements	Complete outcome data	Confounders accounted for	Exposure occurred as intended	Overall score
Cohen et al. [16]	✓	✓	✓	✓	✓	100%
Slattery et al. [17]	✓	✓	✓	✓	✓	100%
Vena et al. [26]	✓	✓	✓	✓	✓	100%

Primary Outcomes

Table 4 summarises the primary outcomes from each study. All OR values reported in this study were obtained by comparing the highest versus the lowest levels of PA. Cohen et al. [16] did not find a significant association between PA and colon cancer risk (OR 1.15, 95% CI 0.47-2.79). In contrast, Slattery et al. [17] found PA to be protective against colon cancer; individuals who undertook the highest level of PA had a reduced risk of developing colon cancer, compared to those who undertook the lowest level (men: OR 0.65, 95% CI 0.44-0.97; women: OR 0.56, 95% CI 0.34-0.93). Slattery et al. [17] also demonstrated a protective effect of PA against rectal cancer (men: OR 0.43, 95% CI 0.26-0.71; women: OR 0.53, 95% CI 0.29-0.96).

**Table 4:** Primary outcomes for each study OR: odds ratio; CI: confidence interval

Author	Colon cancer, OR (95% CI)	Rectal cancer, OR (95% CI)
Cohen et al. [16]	1.15 (0.47-2.79)	
	p > 0.05	
Slattery et al. [17]	Men, 0.65 (0.44-0.97)	Men, 0.43 (0.26-0.71)
	Women, 0.56 (0.34-0.93)	Women, 0.53 (0.29-0.96)
	p ≤ 0.05	p ≤ 0.05
Vena et al. [26]	Men, 1.87 (-)	Men, 0.90 (-)
	p ≤ 0.05	p > 0.05

However, Vena et al. [26] suggested that a higher level of PA could increase the risk of CRC. In this male-only cohort, those who undertook the highest level of PA were more likely to develop colon cancer, compared to those who undertook the lowest level (OR 1.87, p ≤ 0.05; 95% CI not reported). 

Secondary Outcomes

The secondary objective was to determine whether CRC risk varied by type, intensity, and/or frequency of PA. Across the three studies, this could not be determined conclusively. Cohen et al. [16] used a questionnaire assessing leisure-time activities such as strength training, swimming, walking, and cycling, with the highest PA level defined as ≥75 minutes per week of vigorous activity or ≥150 minutes per week of moderate activity. Slattery et al. [17] included both leisure and occupational PA, defining the highest PA level as >1,000 MET minutes per week. Vena et al. [26] assessed only occupational PA, including standing, walking, pulling, and lifting goods. In this study, sedentary work was defined as involving little PA (occasional walking, standing, and lifting small goods), while light work involved lifting goods to 4.5 kg as well as more frequent walking and standing. The highest PA level was defined as working for more than 20 years in occupations involving sedentary or light activity [26]. 

Synthesis of Results

Overall, the three studies presented inconsistent findings. One study found no association between PA and CRC, one reported a protective effect, and one suggested possible harm at higher PA levels. No pooled analysis was undertaken due to methodological and exposure variability.

Certainty of Evidence

Although all studies were assessed as high quality using the MMAT, the overall certainty of the evidence was judged to be low. This reflects inconsistency in study findings, variability in the types and levels of PA examined, and imprecision in effect estimates, including incomplete reporting in one study. 

Discussion

Summary

This systematic review has evaluated the evidence regarding PA and CRC risk among adults aged 65 and above. Three studies were identified, the results of which were conflicting. One study found no association between PA and CRC risk [16]. Another paper suggested PA was protective against CRC, with a reduced risk observed in those who undertook PA at the highest level [17]. The final paper suggested PA was harmful, with an increased colon cancer risk among the most active individuals [26]. Therefore, at present, no definitive conclusion can be drawn with respect to the benefit or risk of PA and the incidence of CRC in this age group.

Strengths and Limitations 

This is the first systematic review to consider the effect of PA on the risk of CRC in older adults. The review has been carried out in accordance with PRISMA 2020 guidelines, and a comprehensive search strategy was applied across four major databases, supplemented by reference list screening, which minimised the likelihood of missing eligible studies. It was also conducted with double-blind eligibility and inclusion screening throughout, reducing the risk of selection or extraction bias. While only three studies were included, they were judged to be of high quality. Importantly, the review addresses a critical gap in the literature by focusing specifically on older adults, a group in whom both cancer incidence and population size are increasing, reinforcing the need for further research to guide public health policy.

The small sample size is a key limitation of this review; of the 3,272 papers that were screened, only three fulfilled the inclusion criteria. The study by Vena et al. [26] was conducted several decades ago, and differences in diagnostic methods, PA assessment, and population characteristics over time may limit its comparability to more recent studies. The included studies also differed in design (one cohort and two case-control), in the populations assessed, and in how PA was measured. Cohen et al. [16] examined leisure-time activities; Slattery et al. [17] combined leisure, occupational, and household activities, while Vena et al. [26] considered only occupational PA. These differences restricted the ability to directly compare or synthesise the results, and likely contributed to the inconsistency in findings across the studies. In addition, while all three studies were judged to be of high methodological quality using the MMAT, limitations in reporting were evident. For example, Vena et al. [26] did not provide confidence intervals for their risk estimates, reducing the precision of their findings.

Slattery et al. [17] and Vena et al. [26] recruited participants from age 30 upwards, and although age-specific data for older adults were extracted where available, these studies were not primarily designed to assess this subgroup. As a result, findings may be influenced by unaccounted confounders such as health status, or reduced precision in estimating associations for adults aged ≥65 years. 

There are also some limitations related to the review process itself. Only studies published in English were included, as there was no access to translation services within the scope of this study; therefore, non-English studies were not eligible, which may have led to the exclusion of some relevant studies. All included studies were conducted in Western countries, which may limit the applicability of findings to other cultural or geographic contexts where lifestyle patterns and CRC risk factors differ. A narrative synthesis was undertaken instead of a meta-analysis, which was appropriate given the heterogeneity in study designs, exposures, and outcome measures. 

Comparisons With Previous Literature

Research investigating the association between CRC and PA is well-established. Several studies have reported a decreased risk of CRC with PA in adults above 50 years [14,15]. Similar results have been reported elsewhere [27-29]. Though these studies do not primarily feature older adults, they are consistent with the findings of Slattery et al. [17].

Studies investigating the association between PA and other cancer types have also been conducted. For example, one study found that women aged 65 years and above who undertook the highest level of PA compared to women of the same age who did not perform any PA, had a lower breast cancer risk (RR: 0.2; 95% CI: 0.05-0.90) [30]. Furthermore, a study involving women aged 55-69 found that those who undertook the highest level of PA compared to those who undertook the lowest level had a lower lung cancer risk (HR: 0.77; 95% CI: 0.64-0.94) [31]. Finally, for endometrial cancer, a study including women aged between 35 and 79 found that compared to those who engaged in the lowest level of PA, women who undertook the highest level had a smaller cancer risk (OR: 0.67; 95% CI: 0.47-0.95) [32]. Though not all of the aforementioned studies focus on adults aged 65 and above, they contribute to the increasing research regarding PA and cancer risk. 

The pathway by which PA confers this protection against CRC is not clear from the literature. However, several theories have been suggested. Immune function may be improved, and inflammation may be reduced through PA; a study conducted in mice found that treadmill running led to lower interleukin-6 (IL-6) levels, a cytokine that plays a role in promoting cancer development [33,34]. Additionally, PA may result in lower carcinogen exposure by shortening transit time through the colon [33]. A faster transit time was reported with jogging (34 hours) and cycling (36.6 hours), compared to at rest (51.2 hours); however, only 10 participants were involved [35]. In contrast, no notable difference was observed in another study between PA (20.9 ± 16.8 hours) and inactivity (24.5 ± 21.8 hours), which included 16 participants during one week with PA and one week without [36]. There is, therefore, conflicting evidence regarding this mechanism in CRC prevention. Protection against CRC may also occur by reducing insulin resistance through PA [33].

Implications for Research

The benefit of PA in lowering the risk of CRC is evident in studies conducted in younger populations. However, increasing age is a risk factor for CRC [33]. Therefore, it is imperative to continue research into cancer prevention measures in older adults. Future research is necessary to determine the association between the risk of CRC and PA in older populations.

Another consideration is whether engaging in regular PA from younger adulthood may confer greater protection against CRC in later life compared to initiating activity at an older age. Further research should therefore aim to distinguish between the long-term cumulative effects of lifelong activity and the benefits of activity undertaken later in life, with respect to CRC risk. 

For adults, WHO currently recommends at least either 75-150 minutes of aerobic PA at a vigorously intense level or 150-300 minutes of aerobic PA at a moderately intense level per week; strength exercises on two or more days of the week are also recommended for additional health benefits [37]. Whether this would be enough to influence the risk of CRC must be investigated. Thus, future research should also assess PA type, frequency, and/or intensity with regard to the risk of developing CRC in older adults. Building on previous knowledge regarding PA and CRC will help clinicians to provide better education about behaviours and lifestyle choices. 

Implications for Practice and Policy

The mixed results in this review raise questions about the role of PA in reducing CRC risk in older individuals. However, there is vast literature to support the positive impact of PA in this age group, including improved mental health and physical function, cardiorespiratory fitness, and lower mortality rates [38,39]. Adding to this is the aforementioned evidence that PA reduces the risk of CRC in other age groups [14,15, 27-29]. PA has also been reported to reduce the risk of other types of cancers [30-32]. Thus, promoting PA among older adults will ultimately lead to better health awareness and outcomes; this form of preventative care will also serve to protect healthcare resources. 

Encouraging PA in this population of adults brings about many challenges, such as the presence of other comorbidities and beliefs regarding PA [20]. One study also found that healthcare professionals were at times unable to give patients advice regarding PA, due to insufficient time, being unfamiliar with guidelines, and not knowing where to find information that could help them better understand how to approach such conversations with patients [40].

It is therefore imperative to consider how beliefs and attitudes regarding PA in this age group can be addressed, and how healthcare professionals can be supported, so they feel more confident and better equipped to have discussions about PA with their patients. Encouraging PA in this age group is likely to involve a multifaceted approach. There may be some benefit in bringing about campaigns at a national level, targeted at adults aged 65 and above, to encourage more PA with the view of protecting them against cancer and other chronic conditions. Additionally, sports organisations could offer more inclusive opportunities that ensure safe participation for older adults, tailored to those both with and without physical limitations.

## Conclusions

PA is widely recognised as a protective factor against several types of cancers, including CRC. While these benefits are well-documented in the general population, there remains a clear knowledge gap regarding the relationship between PA and CRC risk in older adults. This systematic review highlights this, as the limited available evidence remains inconclusive. It is also unclear whether CRC risk varies by the type, intensity, and/or frequency of PA. Older adults may experience age-related physiological changes, comorbidities, and other factors that influence their response to PA. Robust, longitudinal, and standardised studies, including those from non-English and more diverse populations, are therefore needed to clarify these associations. Such research could guide the development of age-appropriate, accessible interventions that promote healthy ageing and reduce the overall cancer burden in this growing population. 
